# Hantavirus Infection Suppresses Thrombospondin-1 Expression in Cultured Endothelial Cells in a Strain-Specific Manner

**DOI:** 10.3389/fmicb.2016.01077

**Published:** 2016-07-19

**Authors:** Svetlana F. Khaiboullina, Sergey P. Morzunov, Stephen C. St. Jeor, Albert A. Rizvanov, Vincent C. Lombardi

**Affiliations:** ^1^Nevada Center for Biomedical ResearchReno, NV, USA; ^2^Department of Genetics, Institute of Fundamental Medicine and Biology, Kazan Federal UniversityKazan, Russia; ^3^Nevada State Public Health Laboratory, University of Nevada, Reno School of MedicineReno, NV, USA; ^4^Department of Immunology and Microbiology, University of Nevada, Reno School of MedicineReno, NV, USA; ^5^Department of Biochemistry and Molecular Biology, University of NevadaReno, NV, USA

**Keywords:** coagulation, HPS, HFRS, THBS1, TSP1, Prospect Hill, Hantaan, Andes

## Abstract

Hantavirus infection is associated with two frequently fatal diseases in humans: Hemorrhagic fever with renal syndrome (HFRS) and hantavirus pulmonary syndrome (HPS). The pathogenesis of hantavirus infection is complex and not fully understood; however, it is believed to involve virus-induced hyperinflammatory immune responses. Thrombospondin-1 (THBS1) is a large homotrimeric protein that plays a putative role in regulating blood homeostasis. Hyperresponsiveness to inflammatory stimuli has also been associated with defects in the THBS1 gene. Our data suggest that hantavirus infection of human umbilical cord vein endothelial cells (HUVEC) suppress the accumulation of THBS1 in the extracellular matrix. Additionally, this suppression is dependent on virus replication, implying a direct mechanism of action. Our data also imply that the pathogenic Andes and Hantaan strains inhibit THBS1 expression while the non-pathogenic Prospect Hill strain showed little inhibition. These observations suggest that a dysregulation of THBS1 may contribute to the pathogenesis of hantavirus infection.

## Introduction

Hantaviruses are negative strand RNA viruses belonging to the family *Bunyaviridae* (Schmaljohn and Nichol, 2001; Schmaljohn, 2007). They are the etiological agent associated with two frequently fatal human illnesses: Hemorrhagic fever with renal syndrome (HFRS) and hantavirus pulmonary syndrome (HPS; Jenison et al., 1995; Enria et al., 2001; Linderholm and Elgh, 2001; Peters and Khan, 2002). Postmortem analyses of HFRS and HPS cases typically reveal high viral loads in endothelial cells, suggesting that the vascular endothelium is a principal site of infection (Zaki et al., 1995). *In vitro* studies have confirmed that human vascular endothelial cells are susceptible to hantavirus infection; however, infection does not produce a visible cytopathic effect (Pensiero et al., 1992; Khaiboullina et al., 2004).

HFRS and HPS are characterized by high fever and myalgia; however, kidney failure and bleeding are typical of HRFS while HPS manifests with pneumonia and cardiovascular dysfunction (Kanerva et al., 1998; Enria et al., 2001; Linderholm and Elgh, 2001). Despite different clinical presentations, decreased platelet counts, and changes in blood chemistry are commonly observed during the early phase of both syndromes (Kanerva et al., 1998; Peters et al., 1999). Disturbed hemostasis is a hallmark of HFRS (Linderholm and Elgh, 2001; Sundberg et al., 2011) as is progressive disseminated intravascular coagulation (DIC) syndrome (Connolly-Andersen et al., 2015); however, severe shock and renal failure tend to associate with more severe illness (Lee et al., 1989; Rasche et al., 2004; Sundberg et al., 2011). HPS commonly manifests with pneumonia and severe cardiovascular dysfunction as a result of progressive leakage of fluid from the blood into the lungs, resulting in pulmonary edema, hypoxia, and circulatory collapse (Enria et al., 2001). Although DIC is not the most prevalent pathology during HPS, laboratory, and histological examinations have revealed the presence of all symptoms of DIC (Zaki et al., 1995). DIC is an acquired disorder in which the proteins that control blood clotting become constitutively activated, resulting in consumption of platelets and conversion of fibrinogen to fibrin (Slofstra et al., 2003). With blood platelets and clotting factors exhausted, internal, and external bleeding typically ensues (Peters et al., 1999). In the normal steady state, endothelial cells control blood homeostasis by continuously expressing several proteins (Cirino et al., 2000; Dugina et al., 2002), many of which interact with thrombospondin-1 (THBS1); a large trimeric glycoprotein secreted by several cell types including endothelial cells, and activated platelets (McPherson et al., 1981; Mast et al., 2000; Resovi et al., 2014; Prakash et al., 2015).

Once secreted, THBS1 suppresses proliferation and migration of endothelial cells, as well as signals their apoptosis (Khan et al., 1996; Lawler and Detmar, 2004). Additionally, THBS1 can induce endothelial cell actin reorganization and focal adhesion disassembly (Greenwood et al., 1998; Goicoechea et al., 2000). Several proteases involved in angiogenesis and endothelial integrity, such as urokinase, plasminogen, matrix metalloproteinase, thrombin, cathepsin, and elastase are also influenced by THSB1 (Hogg et al., 1993; Albo et al., 1997; Bein and Simons, 2000; Liu et al., 2009; Yang et al., 2012; Zhao et al., 2015). Furthermore, THBS1 can directly affect the activity of plasmin and urokinase plasminogen activator, which are important for degradation of fibrin (Silverstein et al., 1986; Hogg et al., 1992; Rabhi-Sabile et al., 1998). Finally, THBS1 controls von Willebrand (vW) factor multimer sizes by reduction of disulfide bonds, which link the vW factor subunits together (Xie et al., 2001). Secretion of THBS1 by endothelial cells can be modulated by proinflammatory cytokines such as TNFα and IL-1, two cytokines that are commonly observed in association with hantavirus infection (McPherson et al., 1981; Lawler and Detmar, 2004; Lopez-Dee et al., 2011). Indeed, several proteins modulated by THBS1 have been implicated in hantavirus pathology. For instance, Bondu and coworkers observed elevated levels of thrombin in severe HPS cases (Saumet et al., 2002; Bondu et al., 2015) and Sadeghi et al. observed TGFB1 to be elevated during the late phase of Puumala hantavirus infection (Sadeghi et al., 2011; Chu et al., 2013). Also, Strandin and colleagues reported that tissue plasminogen activator was strongly upregulated in severe cases of Puumala hantivirus infection (Strandin et al., 2016). Additionally, Davidovich et al. reported a three-fold increase in serum vW in HRFS patients during their oliguric phase (Davidovich et al., 1993). Although the molecular mechanisms involved in hantavirus pathology are not well-understood it is reasonable that THBS1 may play a role in the pathophysiology of hantavirus infection through the interactions with the abovementioned proteins.

Recently, decreased levels of serum THBS1 have been reported in association with HFRS (Liu et al., 2008; Laine et al., 2014). Liu et al. suggested that insufficient production or increased consumption of THBS1 contribute to the impaired integrity of small capillaries in subjects with HFRS (Liu et al., 2008). In the present study, we demonstrate that *in vitro* hantavirus infection of endothelial cells suppresses transcription of THBS1 and its subsequent accumulation in the extracellular matrix. This suppression is dependent on viral replication, but is independent of IL-6 and CCL5 expression by infected cells. Additionally, these observations were strain-specific with the pathogenic Andes (ANDV) and Hantaan (HTNV) strains suppressing THBS1 expression while non-pathogenic Prospect Hill (PHV) strain showed little inhibition.

## Methods

Human umbilical cord vein endothelial cells (HUVEC) and Vero clone E6 (Vero E6) were obtained from Lonza, Inc. (Portsmouth, NH) and American Type Culture Collection (ATCC, Manassas, VA), respectively. HUVEC were grown in MCDB 131 medium, supplemented with human vascular endothelial cell growth factor, hydrocortisone, 2% fetal bovine serum (FBS), human fibroblast growth factor (0.5 mL; 1 μg/mL), ascorbic acid, heparin (0.5 mL; 1 μg/mL), and gentamicin. Cells were used at passages 2–4. Vero E6 cells were grown in DMEM medium containing 20% FBS and gentamicin.

All work with infectious virus was conducted in a BSL-3 facility, in compliance with a Memorandum of Understanding with the University of Nevada, Reno, as well as according to NIH, CDC, and OSHA standards. All virus stocks were propagated on Vero E6 cells. Andes virus strain 23 (ANDV) and Hantaan virus strain 76–118 (HTNV) were a generous gift from Dr. Thomas Ksiazek (CDC, Atlanta, GA), and Prospect Hill virus (PHV) was a generous gift from Dr. Connie Schmaljohn (Fort Detrick, MD). In all experiments, cells were infected at a virus to cell ratio of 3 (MOI of 3) except where indicated. In some experiments, the virus was inactivated with 2 × 10^6^ rads of gamma radiation. Infections were conducted by incubating each respective virus on HUVEC for 1 h at 37°C, 5% CO_2_. The cell monolayers were then washed with HBBS, and new medium was added and the incubation continued for the times indicated. For some experiments, new culture medium was supplemented with 10 ng/mL TNFα, 10 ng/mL TGFβ, 50 ng/ml IL-6, or 12 ng/mL CCL5 (R&D Systems). Mock-infected cells and/or untreated cells were used as controls. All experiments were done in duplicate.

### Immunofluorescence

Hantavirus-infected and mock-infected HUVEC monolayers were fixed with methanol/acetone (3:1), washed three times with PBS (pH 7.4), and incubated with glycine buffer (10 mM glycine in PBS pH 7.4) for 30 min at room temp. Slides were again washed three times with PBS and permeabilized with Triton-X 100 solution (0.1% in PBS pH 7.4) for 30 min at room temp. Slides were next washed three times with PBS and incubated for 30 min at room temp with an appropriate combination of antibodies (summarized in Table 1). Slides were washed again three times in PBS, and incubated with the respective antibody combinations for 30 min at room temp in the dark. Images were captured using a Nikon C1 fluorescent microscope with Easy C1 software.

**Table 1:** **Antibodies**.

**Antibody**	**Concentration**	**Supplier**
Mouse anti-THBS1 mAb	1:400	Sigma
Mouse anti-PUU^*^ mAb	1:100	Dr. Thomas Ksiazek (CDC)
Mouse anti-p-c-Jun mAb	1:100	Santa-Cruz
Rabbit anti-NF-kB	1:100	Santa-Cruz
Rabbit anti-N Protein	1:500	In house
Goat anti-mouse-Alexa 488	1:800	Molecular probes
Donkey anti-rabbit-Alexa 555	1:800	Molecular probes

* *Hantavirus (Puumala) nucleocapsid protein*.

### Western blot

Hantavirus-infected and mock-infected HUVEC were lysed in 200 μL of 0.1% sodium dodecyl sulfate (SDS) solution and standardized using the Better Bradford™ Assay Kit (Pierce, Div. of Thermo Fisher, Waltham, MA). Proteins were electroblotted for 15 min, at 4 mA/cm^2^ onto PVDF membranes (Bio-Rad, Hercules, CA) and blocked for 1 h at room temp with blocking buffer (5% non-fat dry milk in PBS, pH 7.4 and 0.5% Tween 20). Membranes were then washed three times in washing buffer (PBS pH 7.4 and 0.5% Tween 20) and incubated with the respective antibodies in blocking buffer for 12 h at 4°C. Antigen–antibody complexes were identified with goat anti-rabbit-HRP or goat anti-mouse-HRP conjugated antibodies (1:1000 in blocking buffer) and developed using insoluble HRP substrate (Vector Labs, Burlingame, CA) according to the manufacturer's instructions. Western blots were quantitatively measured using Scion Image software version beta 4.0.2.

### Real time PCR

Hantavirus-infected HUVEC and mock-infected cells were collected at selected time points and total RNA was extracted using Trizol reagent (Life Technologies, Carlsbad, CA), according to the manufacturer's instructions. To conduct first strand cDNA synthesis, 3 μL RNA (1 μg total), 1 μL random primer mix (50 μM), and 8.5 μL RNase-free water were combined, denatured at 70°C for 10 min, and chilled at 4°C to anneal the primers. cDNA was then synthesized by adding 1 μL of each dNTP (10 mM; GIBCO, Division of Life Technologies), 1x RT buffer (Promega, Madison, WI), 200 U of MMLV reverse transcriptase (Promega), and 20 U of rRNasin RNAse inhibitor (Promega) in a 20 μL total reaction volume. After incubation for 10 min at 25°C, the reaction proceeded for 1 h at 42°C and was terminated by heating at 95°C for 5 min. cDNA was stored at −20°C until analyzed.

TaqMan analyses were performed using TaqMan minor groove binding probes on an ABI Prism 7000 Sequence Detection System. Each PCR reaction (25 μL) consisted of 1 μL of cDNA, 1õ Platinum qPCR Supermix-UDG (Life Technologies), 200 nM of each primer, and 100 nM of probe. The cDNA of each respective sample was diluted 1:1000 with nuclease free water before TaqMan analyses. The 18S ribosomal gene was used as an endogenous control for all analyses. Standard curves for relative quantification of viral S segment RNA, cellular genes, and 18S RNA were created using serial dilutions of cDNA from infected and uninfected control samples depending on specifics of the experimental design. All TaqMan reactions were performed in triplicate. In TaqMan experiments, TaqMan values of cellular gene mRNA were normalized against TaqMan values for the 18S gene of a corresponding sample. For some experiments, normalized values were expressed as relative values to the same gene expressed in the corresponding control group. The sequences of primers and probe for THBS1 are summarized in Table 2.

**Table 2:** **Sequence and position of primers and probes used for TaqMan**.

**Gene**	**Forward primer**	**Reverse primer**	**TaqMan probe**
ANDV^*^	tcacgccaggacgatttagg (414–433)	ggctttgacctgtgctggaa (472–453)	caattgcttgtggcctt (435–451)
PHV^*^	ggctgacaaagtaaaggcatttc (738–760)	cgtggctcagcctttaggaa (797–778)	tgaccagaaatgtcc (761–776)
HTV^*^	gcagcagttagcctccttggt (787–807)	tgccgctgccgtaagtagt (845–827)	tcctgcaacaaacagg (810–825)
THBS1	gaggcggcctcccctat (1115–1131)	tcaacagtccattcctcgttatttc (1180–1156)	tatcacaacggagttcagtac (1134–1154)
RANTES	cccgcagaggaagcacaa (858–875)	gcgaagatttcccgtaaactttc (919–897)	actacgcgggctgc (861–875)
IL-6	ctgcgcagctttaaggagttc (513–533)	ccatgctacatttgccgaaga (574–554)	cagtccagcctgaggg (537–552)

* *Nucleocapsid “S” segment*.

### Statistical analysis

Data are presented as mean ± *SE*. Statistical analyses were performed using Student's *t*-test for comparisons between individual experimental groups (infected and non-infected). Significance was established at a value of *p* < 0.01.

## Results

### Effect of ANDV, HTNV, and PHV infection and cytokine treatment on THBS1 accumulation in the extracellular matrix of HUVEC

A central dogma of hantavirus pathogenesis is that virus replication leads to endothelial cell dysregulation (Connolly-Andersen et al., 2014). THBS1 is an extracellular matrix protein secreted by endothelial cells and has putative roles in cell adhesion, platelet aggregation, and the regulation of fibrinolysis (Silverstein et al., 1984; Roberts et al., 2010). With this in mind, we sought to determine if hantavirus infection and replication affects THBS1 accumulation in cultured HUVEC. Additionally, we investigated if pathogenic ANDV and HTNV and non-pathogenic PHV differ in their abilities to influence THBS1 accumulation.

**Figure 1:**
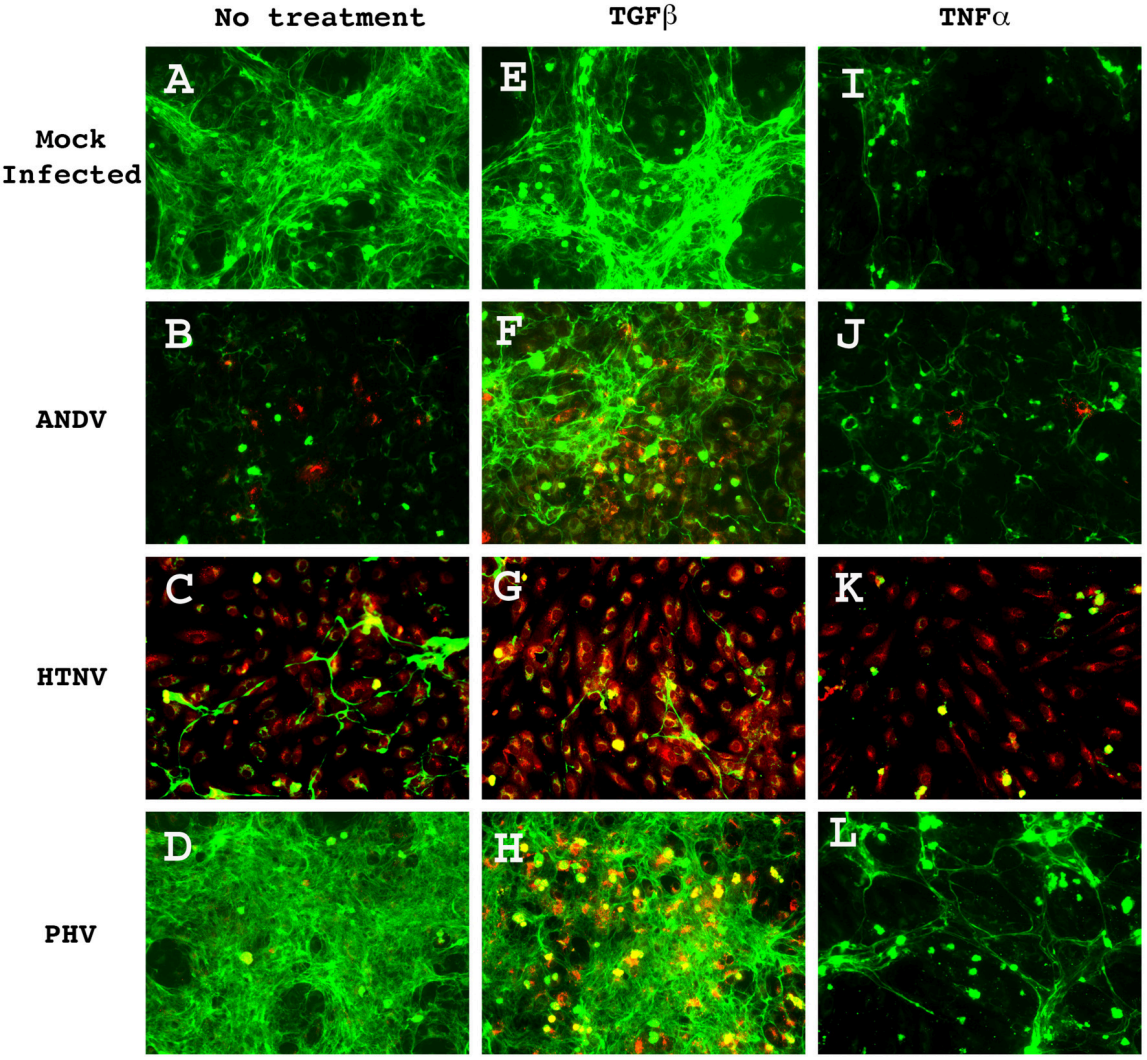
**THBS1 accumulation in the extracellular matrix of HUVEC monolayers infected with pathogenic (ANDV and HTNV) and non-pathogenic (PHV) hantavirus in the presence and absence of TGFβ or TNFα**. Monolayers were infected at MOI 3, with the indicated strains, treated with 10 ng/mL of TGFβ or TNFα, and fixed at 72 h PI. Monolayers were stained with rabbit anti-hantavirus N protein polyclonal antibody followed by donkey anti-rabbit-Alexa 555 secondary (red) and mouse anti-THBS1 mAb followed by a goat anti-mouse-Alexa 488 secondary (green). **(A)** Mock infected, no treatment; **(B)** ANDV infected, no treatment; **(C)** HTNV infected, no treatment; **(D)** PHV infected, no treatment; **(E)** Mock infected, TGFb treated; **(F)** ANDV infected, TGFb treated; **(G)** HTNV infected, TGFb treated; **(H)** PHV infected, TGFb treated; **(I)** Mock infected, TNFa treated; **(J)** ANDV infected, TNFa treated; **(K)** HTNV infected, TNFa treated; **(L)** PHV infected, TNFa treated.

HUVEC monolayers infected with ANDV, HTNV, and PHV were fixed and probed by immunohistochemistry (IHC) for THBS1 accumulation in the extracellular matrix (Figure 1). In mock-infected cells, THBS1 was equally distributed throughout the extracellular matrix (Figure 1A). In contrast, accumulation of THBS1 was greatly reduced in ANDV and HTNV infected HUVEC (Figures 1B,C, respectively). In cells infected with PHV, accumulation of THBS1 was observed throughout the extracellular matrix similar to that observed in uninfected control cells (Figure 1D).

The interactions between THBS1 and transforming growth factor beta (TGFβ) are complex. TGFβ can regulate the expression of extracellular matrix proteins, including THBS1 (Negoescu et al., 1995; Nakagawa et al., 2005). Conversely, discrete sequences of THBS1 have been shown to activate TGFβ (Schultz-Cherry et al., 1995). Therefore, in order to investigate the effect of TGFβ on the accumulation of THBS1 in hantavirus-infected cells we duplicated the above experiment but supplemented the HUVEC culture media with TGFβ. Treatment of mock-infected cells with TGFβ did not significantly affect THBS1 accumulation in the extracellular matrix (Figure 1E); however, when ANDV-infected TGFβ treated and non-treated cells were compared, a significant increase in the accumulation of THBS1 was observed in the TGFβ-treated cells (Figure 1F). Interestingly, when compared to untreated cells, TGFβ did not substantially affect THBS1 accumulation in the extracellular matrix of cells infected with HTNV and PHV (Figures 1G,H, respectively).

Previous reports suggest that secretion of THBS1 by endothelial cells may be modulated by proinflammatory cytokines such as tumor necrosis factor alpha (TNFα; Morandi et al., 1994). To investigate this possibility in the context of hantavirus infection, 10 ng/mL of TNFα was added to the culture media of hantavirus infected, and mock-infected HUVEC. Addition of TNFα reduced THBS1 accumulation in the extracellular matrix of mock-infected HUVEC (Figure 1I). THBS1 no longer appeared equally distributed throughout the monolayer as seen in the mock-infected control without TNFα treatment. THBS1 accumulation in the matrix of ANDV and HTNV infected HUVEC remained low after treatment with TNFα (Figures 1J,K, respectively). Although PHV infection did not affect accumulation of THBS1 in the extracellular matrix of untreated cells, TNFα treatment did inhibited THBS1 accumulation in PHV infected cells similar to that of uninfected controls (Figure 1L).

### Transcriptional activation of THBS1 in hantavirus infected HUVEC

**Figure 2:**
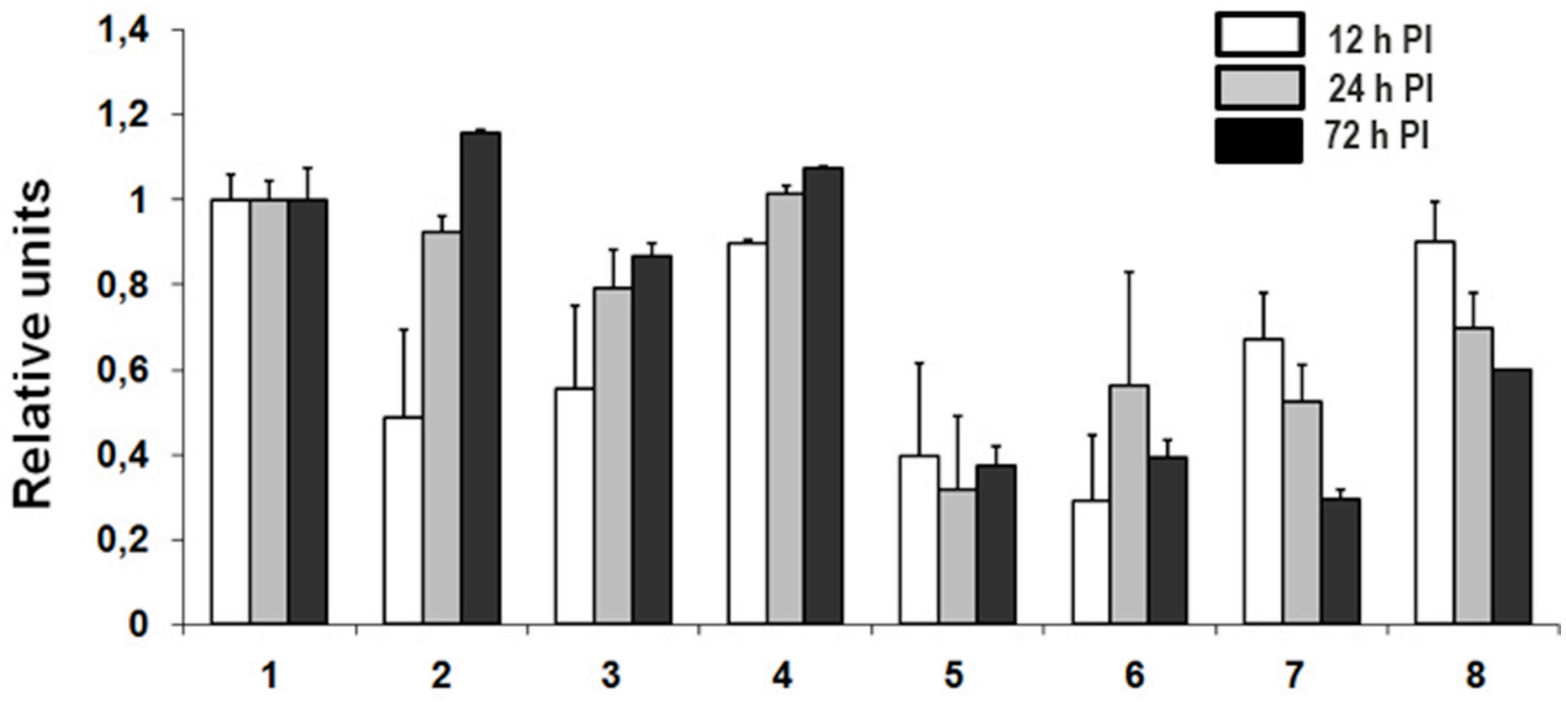
**Transcriptional activation of THBS1 in hantavirus infected HUVEC**. Cells were infected with pathogenic (ANDV and HTNV) and non-pathogenic (PHV) hantaviruses at MOI 3. TNFα was used at 10 ng/mL where indicated. Total RNA was collected at 12, 24, and 72 h PI and used to determine transcription of THBS1 mRNA by TaqMan analysis. Values of THBS1 mRNA were normalized to the 18S mRNA in the corresponding sample. Relative units of THBS1 mRNA were obtained by dividing normalized THBS1 values of infected cells by those of uninfected controls. Each experiment was performed three times and each TaqMan reaction was performed in duplicate. **(1)** Uninfected, **(2)** ANDV infected, **(3)** HTNV infected, **(4)** PHV infected, **(5)** TNFα treated, **(6)** TNFα treated and ANDV infected, **(7)** TNFα treated and HTNV infected, **(8)** TNFα treated and PHV infected.

The effects of hantavirus infection and TNFα treatment on THBS1 transcription were analyzed using TaqMan (Figure 2). Untreated and TNFα-treated (10 ng/mL) HUVEC were infected with ANDV, HTNV, PHV, or mock-infected and total RNA was collected at 12, 24, and 72 h post infection (PI). Pathogenic ANDV and HTNV showed significantly reduced transcriptional activity of THBS1 in HUVEC 12 h PI when compared to mock-infected cells (*p* < 0.01; Figures 2.2, 2.3). Transcription of THBS1 RNA in ANDV and HTNV infected cells returned to the levels of mock-infected cells at 72 h PI (Figures 2.2, 2.3). In HUVEC infected with non-pathogenic PHV, THBS1 RNA levels did not differ significantly from that of mock-infected HUVEC at all time points PI (Figure 2.4). TNFα treatment significantly suppressed THBS1 RNA levels in mock-infected HUVEC at 12, 24, and 72 h PI compared to untreated cells (*p* < 0.01; Figure 2.5). THBS1 RNA levels were reduced in TNFα-treated HUVEC infected with pathogenic ANDV and HTNV similar to that of uninfected TNFα-treated controls at all time points (*p* < 0.01; Figures 2.6, 2.7). Interestingly, THBS1 RNA levels in PHV-infected HUVEC treated with TNFα did not differ significantly from that of PHV-infected cells without TNFα treatment at 12 h PI (Figures 2.4, 2.8). However, THBS1 RNA levels were significantly lower in PHV-infected HUVEC treated with TNFα as compared to that in PHV-infected cells without TNFα treatment at 24 and 72 h PI (*p* < 0.01; Figures 2.4, 2.8).

### Effect of hantavirus replication on THBS1 accumulation in the extracellular matrix of HUVEC

**Figure 3:**
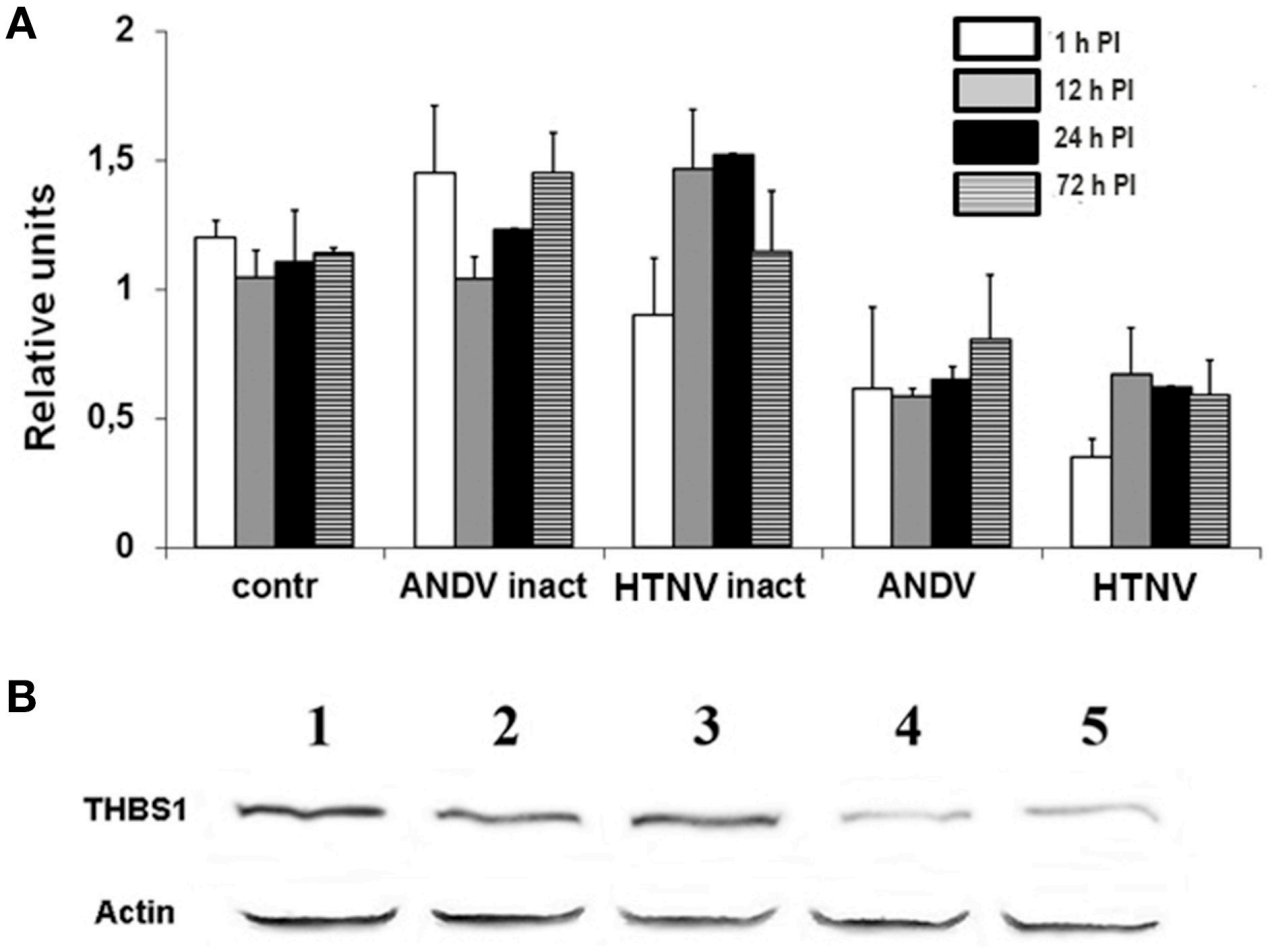
**Effect of hantavirus replication on THBS1 transcriptional activity and protein expression in HUVEC**. Cells were inoculated with gamma radiation inactivated or infectious ANDV and HTNV at MOI of 1. Total RNA was collected and used to evaluate THBS1 transcription **(A)**. Values of THBS1 were normalized to the corresponding values of 18S RNA. Relative units were calculated by dividing normalized values in infected group by normalized values in mock-infected control group. To determine the effect of hantavirus replication on THBS1 protein accumulation in HUVEC **(B)**, Total proteins collected from HUVEC inoculated with gamma radiation inactivated and infectious ANDV and HTNV (MOI 1; 72 h PI) were separated by SDS-PAGE and transferred onto the PVDF membrane. Membranes were probed with anti-THBS1 mAb followed by anti-mouse HRP-conjugated secondary antibody. 1. Mock infected, 2. Inactivated ANDV, 3. Inactivated HTNV, 4. Infectious ANDV, and 5. Infectious HTNV.

To assess if an active infection is required for the accumulation of THBS1 in the extracellular matrix, HUVEC were inoculated with ANDV and HTNV, as well as the same viruses inactivated by gamma radiation. Total RNA was then collected at selected time points after inoculation (1, 24, 48, and 72 h PI) for evaluation using TaqMan. ANDV and HTNV S segment RNA was measured to confirm hantavirus replication (data not shown). Transcriptional activity of THBS1 in HUVEC inoculated with inactivated ANDV or HTNV did not differ significantly from that of mock-infected HUVEC at all selected time points (1, 24, 48, and 72 h PI; Figure 3A). However, the levels of THBS1 RNA were significantly lower in HUVEC inoculated with competent ANDV or HTNV at each selected time point PI when compared to cells inoculated with inactivated ANDV or HTNV (*p* < 0.01; Figure 3A). The effects of competent and gamma-irradiated ANDV and HTNV on the expression of THBS1 in HUVEC were also analyzed using Western blot (Figure 3B). THBS1 expression in HUVEC inoculated with gamma-irradiated ANDV or HTNV did not differ from that of mock-infected HUVEC (Figure 3B). In contrast, cells infected with competent ANDV or HTNV showed clear reduction in THSB1 expression (Figure 3B).

### Effect of hantavirus replication on c-Jun activation and NF-kB expression

**Figure 4:**
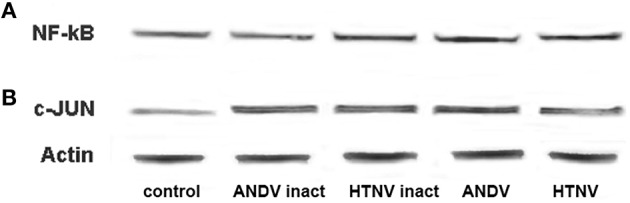
**Effect of hantavirus replication on NF-kB expression and c-Jun activation**. Total proteins collected from HUVEC inoculated with gamma radiation inactivated and infectious ANDV and HTV (MOI of 1; 72 h PI) were separated in SDS-PAGE and transferred onto the PVDF membrane. Membranes were probed with anti-NF-kB **(A)** or anti-phospho-c-Jun **(B)** mAbs followed by anti-mouse-HRP conjugated secondary antibody.

Gamma-irradiation hinders virus replication, but preserves viron integrity, thus allowing viral proteins to interact with cellular proteins in the absence of virus replication. Additionally, it has been shown that the expression of THBS1 can be inhibited by activation of the c-Jun member of the AP-1 family of transcription factors (Bohmann et al., 1987; Curran and Franza, 1988), as well as NF-kB activation (Cinatl et al., 2000). With this in mind, we sought to determine if viral replication was required to activate these transcription factors. We observed similar levels of NF-kB expression and c-Jun activation in HUVEC inoculated with inactivated or competent ANDV and HTNV (Figures 4A,B, respectively at 72 h PI). These observations suggest NF-kB expression and c-Jun activation are independent of virus replication and, therefore, are unlikely to represent the mechanism of THSB1 inhibition by hantaviruses.

### Activation of CCL5 and IL-6 expression in HUVEC infected with ANDV, HTNV, and PHV

Hantaviruses may affect the expression of THBS1 in endothelial cells by indirect mechanisms such as by promoting cytokine and chemokine production. At least two cytokines, TNFα and interleukin-1 (IL-1), have been shown to suppress THBS1 expression by endothelial cells (Morandi et al., 1994). However, we as well as others have shown that hantavirus infection does not activate TNFα or IL-1 expression in endothelial cells (Sundstrom et al., 2001; Geimonen et al., 2002; Khaiboullina et al., 2004). Nevertheless, we have previously shown that hantaviruses promote the expression of CCL5 in HUVEC (Khaiboullina et al., 2004). During the course of these previous studies, we did not observe any changes in IL-6 gene expression in hantavirus-infected cells at early time points (3 and 12 h PI; Khaiboullina et al., 2004). We did however observe that the transcriptional regulator nuclear factor for interleukin-6 (CCAAT/Enhancer Binding Protein), which activates IL-6 gene expression (Akira et al., 1990; Kinoshita et al., 1992), was upregulated in infected cells (Geimonen et al., 2002; Khaiboullina et al., 2004) suggesting that IL-6 expression might be activated at a later time point. Based upon these data, we hypothesized that hantavirus-induced CCL5 and IL-6 expression may lead to a change in the accumulation of THBS1 in HUVEC. We therefore infected HUVEC with ANDV, HTNV, and PHV and collected total RNA at selected time points (12, 24, and 72 h PI) for TaqMan analysis and collected cell culture media supernatants for cytokine analysis by ELISA. Mock-infected cells were used as controls.

**Figure 5:**
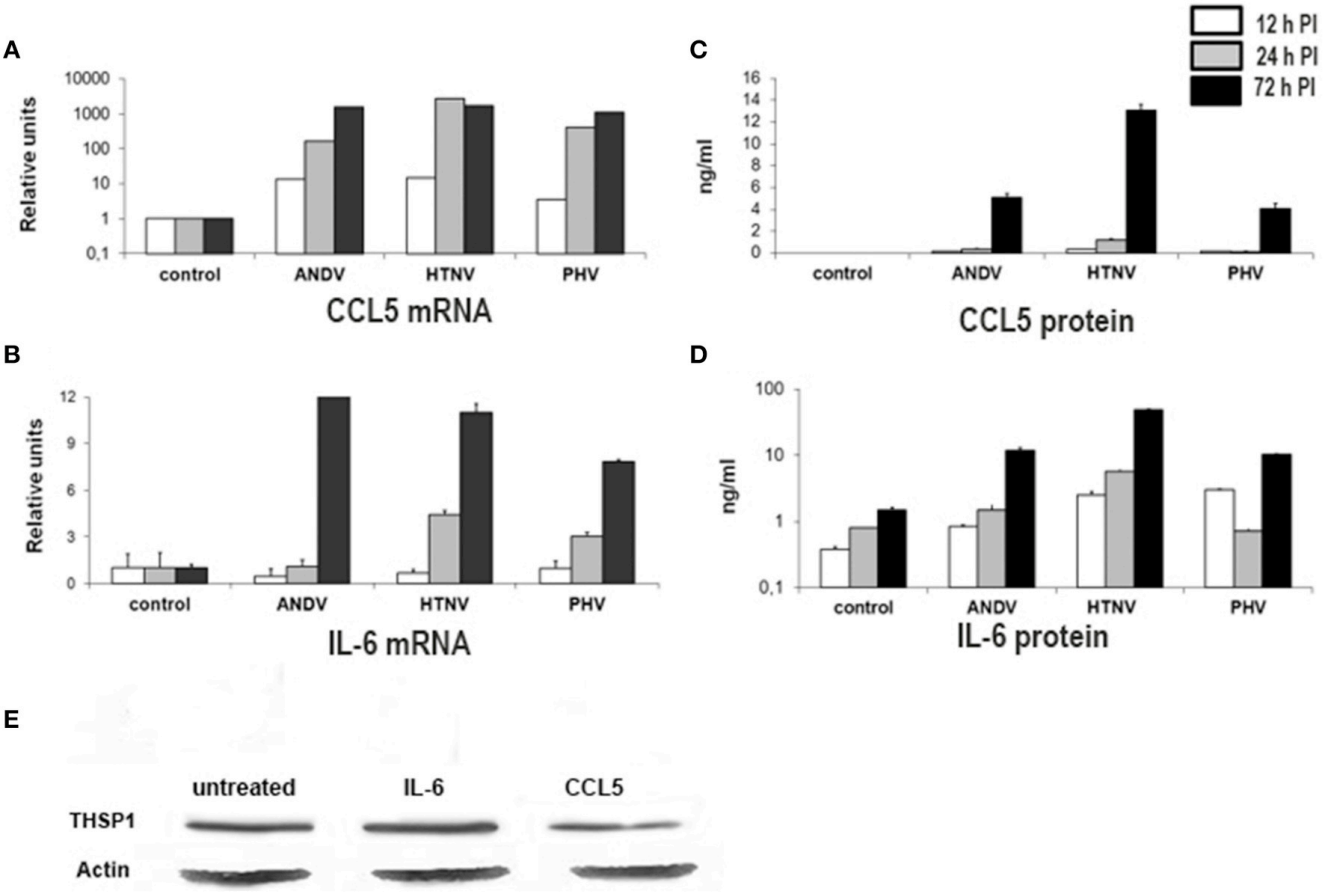
**Transcriptional activity and secretion of CCL5 and IL-6 in hantavirus-infected cells**. Total RNA and cell culture supernatants were collected from hantavirus infected cells at 12, 24, and 72 h PI. Transcription of CCL5 **(A)** and IL-6 **(B)** was analyzed using TaqMan. Levels of CCL5 **(C)** and IL-6 **(D)** in cell culture media were determined using ELISA. Effect of IL-6 and CCL5 on THBS1 expression in HUVEC by Western blot **(E)**. Total proteins were collected from cells either untreated, treated with 50 ng/mL IL-6 or 12 ng/mL of CCL5 for 72 h. Proteins were separated by SDS-PAGE, transferred onto PVDF membrane and probed with anti-THBS1 mAb antibodies followed by anti-mouse-HRP conjugated secondary antibody.

When compared to mock-infected cells, HUVEC infected with ANDV, HTNV, and PHV were observed to upregulate levels of CCL5 RNA at 12, 24, and 72 h PI (Figure 5A). Additionally, transcription of IL-6 was significantly increased in HTNV and PHV-infected HUVEC at 24 h PI and increased for all viruses at 72 h PI, when compared to mock-infected cells (*p* < 0.01; Figure 5B). Also, the levels of CCL5 and IL-6 were significantly increased in culture supernatant of HUVEC infected with ANDV, HTNV, and PHV at 72 h PI when compared to mock-infected cells (*p* < 0.01; Figures 5C,D, respectively).

During the course of these experiments, we observed that the maximum concentration of CCL5 and IL-6 found in supernatants of hantavirus-infected HUVEC cultures was 12 and 50 ng/mL, respectively. Therefore, we used these concentrations to treat uninfected HUVEC in order to assess the effect of CCL5 or IL-6 on THBS1 accumulation in the extracellular matrix. No changes in THBS1 accumulation were observed upon treatment of HUVEC with CCL5 or IL-6 when compared to untreated controls, as determined by Western blot analysis (Figure 5E), suggesting that the effect was not strictly the result of these inflammatory cytokines.

## Discussion

Although it has been suggested that the high viral loads observed in the endothelial cells of those with HFRS and HPS is related to disease pathogenesis (Yi et al., 2014; Bellomo et al., 2015), the mechanisms of pathogenesis remains unknown. Hantaviruses are not cytopathic *in vitro* (Pensiero et al., 1992; Sundstrom et al., 2001; Khaiboullina et al., 2004), and no pathological evidence of virus-induced cell death has been reported in tissues from fatal hantavirus cases (Zhang et al., 1987; Zaki et al., 1995). The absence of virus-induced cytopathicity *in vitro* is in stark contrast with the severe vascular disorders observed during HPS and HFRS (Sargianou et al., 2012; Manigold and Vial, 2014). For this reason, it has been suggested that hantavirus pathology more likely involves a dysregulation of the infected endothelial cell's ability to support their primary function as a blood-tissue barrier (Connolly-Andersen et al., 2014).

In this report, we have demonstrated the *in vitro* suppression of THBS1 transcription in endothelial cells through hantavirus infection. We additionally show that hantavirus infection reduces THBS1 protein accumulation in the extracellular matrix of endothelial cells. Importantly, the inhibitory effects of hantavirus infection were strain specific, whereby, the pathogenic hantaviruses ANDV and HTNV were more effective in suppressing the extracellular matrix accumulation of THBS1. On the other hand, the effects of the non-pathogenic PHV were less pronounced. Additionally, suppression of THBS1 was dependent on virus replication and did not require the presence of CCL5 or IL-6. Our data suggests that hantavirus infection directly influences the expression and accumulation of THBS1, an endothelial cell protein with putative roles in cell adhesion, platelet aggregation, and the regulation of fibrinolysis (Silverstein et al., 1984; Roberts et al., 2010). These data support a mechanism of disease pathogenesis characterized by decreased expression of THBS1 in infected endothelial cells, which in turn leads to a perturbation of normal vascular integrity, and possibly hemostasis, which ultimately contribute to the severe vascular disorders characteristic of pathogenic hantavirus pathology (Adams, 1997; Sargianou et al., 2012; Manigold and Vial, 2014). This pathology may include loss of renal function, capillary leakage, and thrombocytopenia (Thakar et al., 2005; Garg et al., 2011; Sargianou et al., 2012; Vaheri et al., 2013; Latus et al., 2015).

Among the many functions of THBS1, three are of particular interest in relation to hantavirus pathogenesis: first, THBS1 controls vW factor multimer sizes (Xie et al., 2001; Pimanda et al., 2004). vW factor mediates adhesion of platelets at sites of vascular injury; however, only the very large vW factor multimers are effective in promoting platelet adhesion (Sadler, 1998; Xie et al., 2001). THBS1 can reduce intersubunit disulfide bonds of vW factor leading to formation of smaller multimers that have significantly lower activity for platelet aggregation, thus preventing thrombosis (Thakar et al., 2005) and protecting the integrity of vascular endothelium (Xie et al., 2001).

Second, THBS1 suppresses plasminogen activator inhibitor, which allows tissue plasminogen activator and urokinase plasminogen activator to convert plasminogen into plasmin, the principal enzyme responsible for fibrin degradation in the process of fibrinolysis (Silverstein et al., 1986; Hogg et al., 1992; Rabhi-Sabile et al., 1998). Accordingly, an absence of THBS1 and a decrease in fibrinolysis, may lead to excessive coagulation and consumption of clotting factors (Lee, 1987). However, THBS1 is also a slow tight-binding inhibitor of plasmin (Hogg et al., 1992). Therefore, in one context THBS1 may increase platelet aggregation, but in another context prevents blood clots from developing and becoming problematic. In fact, THBS1 released from activated platelets participates both in the formation as well as the resolution of the fibrin clot (Adams, 1997). Accordingly, THBS1 likely influences the balance between procoagulation and anticoagulation proteins, in a context-dependent manner, thus regulating blood flow.

Finally, Bauer et al. reported that circulating THBS1 blocks endothelial-dependent decreases in blood pressure by limiting the production of the diffusible vasodilator nitric oxide (NO; Bauer et al., 2010). Consistent with this observation, and in the context of this study, Liu et al., reported decreased circulating levels of THBS1 in subjects with HFRS (Liu et al., 2008). These data offer an alternative mechanism that is not directly related to the coagulation cascade. By suppressing THBS1 expression in endothelial cells, hantaviruses may affect normal blood flow by decreasing thrombolysis, promoting platelet aggregation, and increasing vascular permeability. However, it should be noted that blood homeostasis is a dynamic process, involving many proteins. THBS1 is known to interact with as many as 50 different proteins (Resovi et al., 2014), and therefore, the precise mechanism of THBS1's involvement may be indirect or through undetermined interactions.

In a previous study to characterized ANDV infection in its native host, Peromyscus maniculatus, Spengler et al., reported that the largely anti-inflammatory cytokine TGFβ was markedly upregulated (Spengler et al., 2013). They further speculate that this observation may explain why ANDV infection in not associated with any clinical signs of pathology in its native host. We observed a paucity of THBS1 accumulation in the extracellular matrix of ANDV-infected HUVEC, however, when ANDV-infected cells were treated with TGFβ, a significant increase in the accumulation of THBS1 was observed (Figure 1F). We also observed this effect to be suppressed in the presence of the proinflammatory cytokine TNFα. Indeed, it has been reported that TNFα antagonizes the activities of TGFβ and is believed to play an essential role in maintaining the stability of extracellular matrix proteins (Verrecchia et al., 2000; Verrecchia and Mauviel, 2004).

As shown in Figure 2, treatment of HUVEC with TNFα also suppressed THBS1 gene transcription in uninfected endothelial cells. In fact, THBS1 distribution in the extracellular matrix of TNFα-treated and uninfected endothelial cells appear similar to that of untreated endothelial cells infected with pathogenic hantaviruses (Figure 1). Several signal transduction pathways are activated by TNFα that can affect THBS1 accumulation, including c-Jun and NF-κB transcription activation factors (Manna et al., 1998; Rahman, 2000). For instance, Kim and Hong reported that decreased THBS1, in response to the inflammatory stimuli phorbol 12-myristate 13-acetate, was regulated by c-Jun (Kim and Hong, 2000). Our data suggest that hantavirus infection activates c-Jun; therefore, it is possible that TNFα may synergizes hantavirus-triggered activation of transcription factors including those involved in THBS1 accumulation. Although TNFα is not directly produced by endothelial cells, an increase in serum TNFα has been reported in the blood of hantavirus infected cases (Krakauer et al., 1995).

While non-pathogenic PHV suppressed the accumulation of THBS1 protein in endothelial cells, the effect was less pronounced when compared to pathogenic strains. Similarly, PHV was not a strong suppressor of THBS1 gene transcription when compared to pathogenic hantaviruses. These data suggest that the non-pathogenic hantavirus have less effect on the expression of endothelial cell proteins involved in the control of blood homeostasis than pathogenic hantaviruses. These observations potentially identify a mechanism for the different pathologies of different hantavirus strains.

Finally, our data suggest that hantavirus replication is required for the suppression of THBS1 accumulation. Although CCL5 and IL-6 upregulation did not suppress THBS1 accumulation, it's possible that c-Jun may be involved. It has been shown for several other viruses that viral proteins can modulate cell protein transcription by interacting with transcription activation factors. For example, the major early regulatory protein large T-Ag, of John Cunningham virus (JC virus), can interact with AP-1 transcription activation proteins (Kim et al., 2003). Also, hepatitis C virus has been shown to activate c-Jun N-terminal kinase and p38 mitogen-activated protein (MAP) kinase (Erhardt et al., 2002). The mechanisms of transcription factor activation by hantaviruses remain to be determined; however, it is possible that viral proteins and/or RNA-protein complexes may be involved as suggested by the activation of c-Jun.

In summary, our data suggest that pathogenic Andes and Hantaan virus infection of endothelial cells suppress the accumulation of THBS1 in the extracellular matrix while the non-pathogenic Prospect Hill strain display little inhibition. THBS1 interacts with numerous members of the coagulation cascade, suggesting a mechanism of pathology. However, the pathophysiology of hantavirus infection is complex and involves not only the dysregulation of coagulation, hyperinflammatory immune responses likely play an important role, that may be independent of coagulation. Although the involvement of THBS1 suggests a potential disease mechanism, further studies will be required to fully understand its involvement in the disease process.

## Author contributions

SK and SS conceived of the project, SK AR, and SM conducted experiments, SK and VL wrote the manuscript.

### Conflict of interest statement

The authors declare that the research was conducted in the absence of any commercial or financial relationships that could be construed as a potential conflict of interest.
